# Mechanisms of Ferroptosis-Related Genes in Gallbladder Cancer Based on Bioinformatics Analysis

**DOI:** 10.1007/s12033-024-01159-w

**Published:** 2024-04-18

**Authors:** Miao Li, Hang Shi, Jing Dong, Ning Lu, Jinjie Lou, Yangbo Xu

**Affiliations:** Department of Oncology, Ningbo TCM Hospital Affiliated to Zhejiang Chinese Medical University (Ningbo Hospital of Traditional Chinese Medicine), No. 819, Liyuan North Road, Ningbo, 315000 Zhejiang China

**Keywords:** Ferroptosis, Gallbladder cancer, Immune cell infiltration, Diagnostic, Bioinformatics

## Abstract

Gallbladder Cancer (GBC) is a lethal malignancy with limited treatment options and poor prognosis. Recent studies have emphasized the role of ferroptosis, a regulated form of cell death, in various cancers, including GBC. We applied bioinformatics methodologies on four GBC datasets to identify differentially expressed genes (DEGs). An intersection of DEGs from the four datasets with ferroptosis and GBC-associated genes was done to identify key ferroptosis-related genes in GBC. GSVA pathway enrichment analysis and immune cell infiltration assessment were conducted to explore their functional roles and interactions. Seven ferroptosis-related genes, EZH2, MUC1, PVT1, GOT1, CDO1, LIFR, and TFAP2A, were identified to be related to GBC. These genes were associated with vital signaling pathways like the G2/M checkpoint and DNA repair and showed significant correlations with immune cell infiltration in GBC. Receiver Operating Characteristic (ROC) curve analysis revealed their high diagnostic potential, with Area Under the Curve (AUC) values ranging from 0.796 to 0.953. Our findings underscore the pivotal role of ferroptosis in GBC and the potential of ferroptosis-related genes as diagnostic biomarkers. This study lays a foundation for further research into ferroptosis-based therapeutic strategies for GBC.

## Introduction

Gallbladder cancer (GBC) is a malignancy that predominantly affects the biliary tract, accounting for more than 50% of all biliary tract malignancies [1], with an insidious onset and a generally poor prognosis [2]. The complex biology of GBC, along with its late presentation and resistance to conventional chemotherapy, render it one of the most lethal cancers worldwide [3]. Despite advancements in diagnostic techniques and therapeutic strategies, most patients have already been at an advanced stage when diagnosed, and only approximately 25% of which have the opportunity for surgery [4]. Moreover, 60–70% of patients suffer recurrence after surgery, with a 5-year survival rate of only approximately 5–15% [5]. Therefore, it is necessary for a deeper understanding of the underlying molecular mechanisms of GBC and the identification of novel prognostic markers.

Recently, ferroptosis, a novel form of regulated cell death characterized by the iron-dependent accumulation of lipid peroxides, has garnered significant attention in the realm of oncology [6]. Distinguished from traditional apoptosis, necrosis, and autophagy, ferroptosis is implicated in various physiological and pathological processes, including cancer [7]. Increasing evidences suggest that modulating ferroptosis could offer a promising approach in cancer treatment [8]. Therapy-resistant cancer cells are exquisitely vulnerable to ferroptosis [7]. Ferroptosis plays a critical role in killing tumor cells and suppressing tumor growth, and it has been considered as a cause of breast cancer, non-small cell lung cancer, and other tumor-causing cell deaths [9]. However, the specific role and underlying mechanisms of ferroptosis in GBC are still relatively uncharted territory, necessitating further exploration.

In the wake of the genomics era, bioinformatics has emerged as an indispensable tool for exploring complex biological systems and processes, including the pathogenesis of diseases like cancer [10]. High-throughput sequencing technologies and bioinformatics analysis have revolutionized our understanding of disease mechanisms at the molecular level, facilitating the identification of disease-specific biomarkers and therapeutic targets [11]. Additionally, the ongoing enhancement of medical research databases, coupled with the exploration of diverse sequencing data, has ignited a global wave of investigation into molecular evolution and gene function at the genomic level [12].

In this study, we delved into the role of ferroptosis in GBC by bioinformatics methodologies. Our main objective was to identify ferroptosis-related genes that are differentially expressed in GBC and investigate their potential diagnostic roles. Further, we sought to analyze the relationship between these genes and key signaling pathways and evaluate their implications on immune cell infiltration within GBC. Collectively, our investigation underscores the significance of ferroptosis in the pathogenesis and diagnosis of GBC, and illuminates potential avenues for therapeutic intervention.

## Material and Methods

### Data Collection and Preprocessing

Gene expression data of GBC was downloaded from four datasets GSE74048, GSE76633, GSE139682, and GSE202479 in the Gene Expression Omnibus (GEO) database (https://www.ncbi.nlm.nih.gov/geo/). A total of 35 GBC patient samples and healthy samples were included in these datasets. These datasets were subjected to quantile normalization and log2 transformation using the R4.2.2 package “limma”. After merging data in these datasets, we employed the “Combat” algorithm from the “sva” package in R4.2.2 to remove the batch effect. Principal component analysis (PCA) was applied to examine the batch effect before and after removal. In addition, ferroptosis-related genes were collected from the FerrDb database (http://www.zhounan.org/ferrdb/current/). According to the keyword “gallbladder carcinoma”, the GBC-related genes were screened from DisGeNET (https://www.disgenet.org/) and GeneCards (https://www.genecards.org/).

### Differential Expression Analysis

The “limma” package in R4.2.2 was performed in differential gene expression analysis between GBC tumor samples and normal controls. Genes with an adjusted *P* < 0.05 and |log_2_FC (fold change)| >1 were considered differentially expressed genes (DEGs). The volcano plot and heatmap of DEGs were constructed using the “ggplot2” package in R4.2.2.

### Ferroptosis-Related Genes in GBC

The intersections among DEGs, ferroptosis-related genes, and GBC-related genes were further screened using the “VennDiagram” package in R4.2.2.

### Gene Set Variation Analysis (GSVA)

The “h.all.v2022.1.Hs.symbols.gmt” was downloaded from MSigDB (https://www.gsea-msigdb.org/gsea/msigdb/). GSVA was performed using the “GSVA” package in R4.2.2 to transform gene-level data into pathway-level data. Then, we conducted a *t*-test to identify pathways with a significantly different enrichment score between GBC and normal samples (*P* < 0.05). The association between the intersecting genes and the screened pathways was also explored using Pearson correlation.

### Immune Infiltration Analysis

The “CIBERSORT” algorithm (https://cibersortx.stanford.edu/) was applied to infer the proportions of infiltrating immune cells from gene expression data. The immune cell types with zero values across all samples were excluded. Differential analysis was performed to compare the infiltration levels of immune cell types between GBC and normal samples (*P* < 0.05).

### Diagnostic Prediction Assessment

We performed univariate logistic regression analysis to assess the predictive power of the identified ferroptosis-related genes in GBC. The performance of these genes as diagnostic markers was assessed using Receiver Operating Characteristic (ROC) curves and quantified by the Area Under the Curve (AUC) using the “pROC” package in R4.2.2 (R Foundation for Statistical Computing, Vienna, Austria).

## Results

###  Identification of Ferroptosis-Related Genes in GBC

**Fig. 1:**
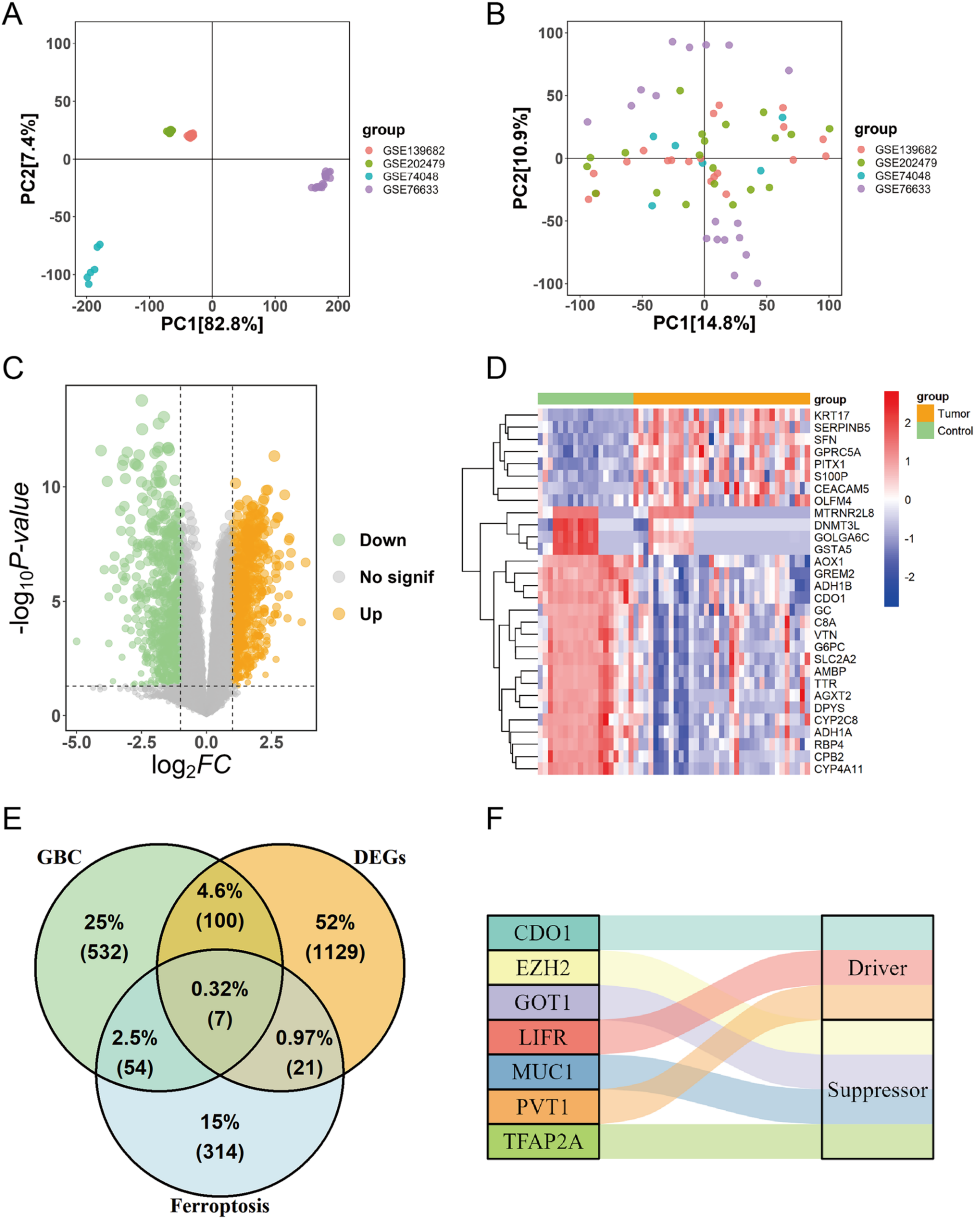
Identification of differentially expressed genes (DEGs) in gallbladder cancer (GBC) and their correlation with ferroptosis-related genes. (**A**) Principal component analysis (PCA) of the four GBC datasets before batch effect removal. (**B**) PCA of the four GBC datasets after batch effect removal. (**C**) Volcano plot showing the DEGs associated with GBC. Orange dots indicate upregulated genes and green dots represent downregulated genes. (**D**) Heatmap showing the expression patterns of the top 30 DEGs between tumor samples and normal controls. (**E**) Venn diagram displaying the overlap among 396 ferroptosis-related genes, 693 GBC-associated genes, and 1257 DEGs, with identification of seven common genes: EZH2, MUC1, PVT1, GOT1, CDO1, LIFR, and TFAP2A. (**F**) The role of the seven intersecting genes in the regulation of ferroptosis

Four GBC datasets (GSE74048, GSE76633, GSE139682, and GSE202479) were merged and the batch effect was removed. Notably, a distinct batch effect was observed among the four datasets, as evidenced by the disparate distribution of PCA dimension reduction before the data were merged (Fig. 1A). Post-removal of batch effects, the distribution of data in four datasets on PCA dimension reduction was considerably uniform (Fig. 1B). In these datasets, we identified 1257 DEGs associated with GBC, including 580 upregulated and 677 downregulated genes. These DEGs were visually represented in a volcano plot (Fig. 1C). A heat map was used to show the expression patterns of the top 30 DEGs between GBC tumor samples and normal controls (Fig. 1D).

We further screened 396 ferroptosis-related genes from FerrDb and 639 GBC-associated genes from DisGeNET and GeneCards. An overlap was identified among the 396 ferroptosis genes, 693 disease-associated genes, and 1257 DEGs. Seven genes were common among these datasets: EZH2, MUC1, PVT1, GOT1, CDO1, LIFR, and TFAP2A (Fig. 1E). Interestingly, among these, PVT1, CDO1, and LIFR were found to be ferroptosis-driving regulators, while EZH2, MUC1, GOT1, and TFAP2A were identified as ferroptosis suppressor regulators (Fig. 1F).

### GSVA Pathway Enrichment Analysis

**Fig. 2:**
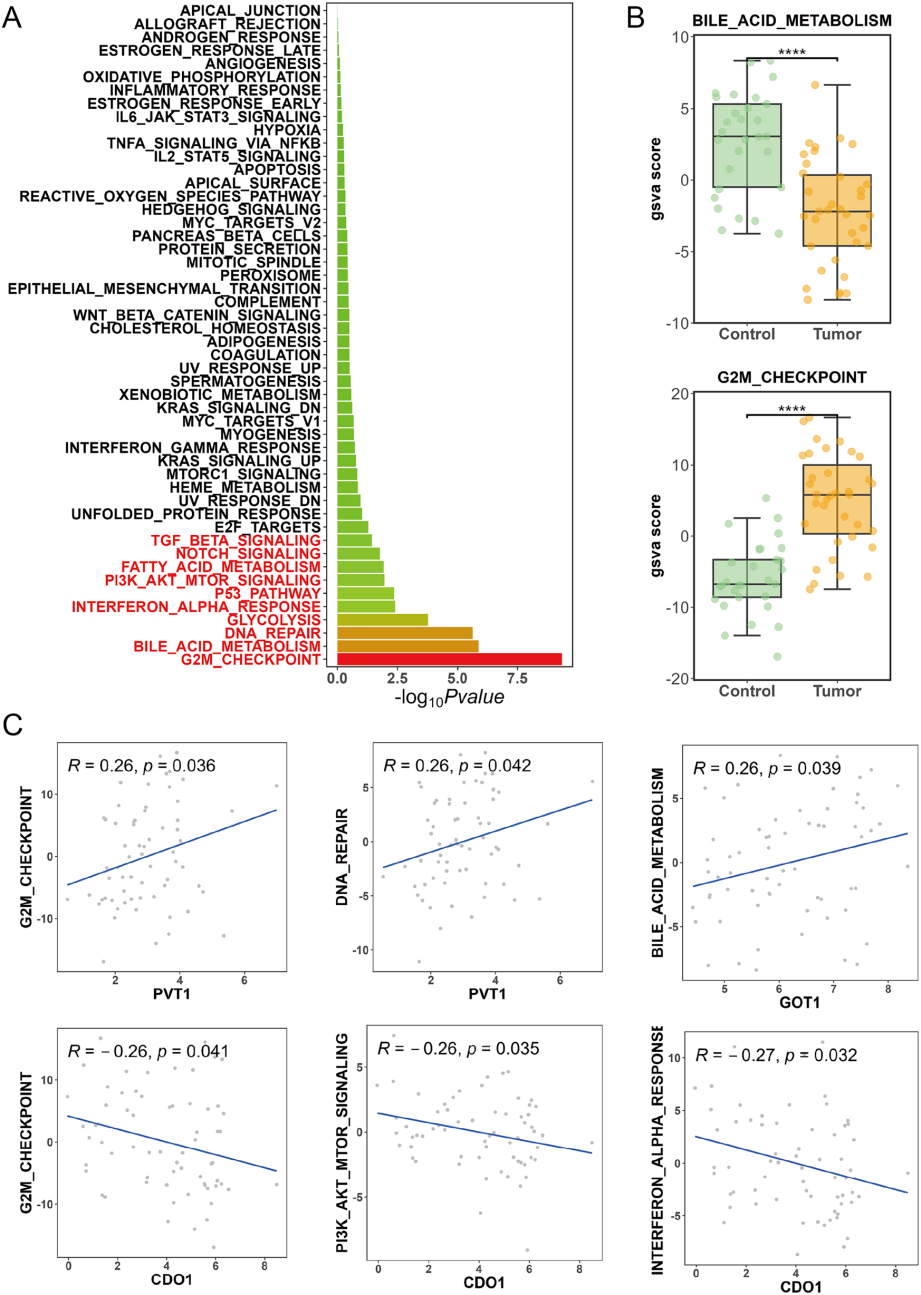
Gene set variation analysis (GSVA) pathway enrichment analysis. (**A**) Bar chart shows the top ten signaling pathways with significant differences in scores. (**B**) Box plots indicate the difference of the G2/M checkpoint and bile acid metabolism in the tumor and control groups; ****P < 0.0001. (**C**) Scatter plots illustrate the correlation between the expression levels of intersection genes and pathway scores

We performed a *t*-test statistical analysis on the GSVA pathway enrichment score matrix. Ten signaling pathways were identified a significant difference in scores between the GBC tumor group and the control group with threshold of *P* < 0.05. The detailed information on these ten pathways is presented in Table 1 and Fig. 2A. Among these pathways, the G2/M checkpoint, bile acid metabolism pathways, and DNA repair exhibited particularly significant score differences (*P* < 0.0001, Fig. 2A). Besides, compared to the control group, bile acid metabolism was inhibited while the G2/M checkpoint was activated in the tumor group (Fig. 2B).

**Table 1:** Differences in access scores

Pathway	Group1	Group2	*P*-value	Significance
G2M_CHECKPOINT	Control	Tumor	4.50E − 10	****
BILE_ACID_METABOLISM	Control	Tumor	1.30E − 06	****
DNA_REPAIR	Control	Tumor	2.30E − 06	****
GLYCOLYSIS	Control	Tumor	0.00017	***
INTERFERON_ALPHA_RESPONSE	Control	Tumor	0.004	**
P53_PATHWAY	Control	Tumor	0.0044	**
PI3K_AKT_MTOR_SIGNALING	Control	Tumor	0.011	*
FATTY_ACID_METABOLISM	Control	Tumor	0.012	*
NOTCH_SIGNALING	Control	Tumor	0.017	*
TGF_BETA_SIGNALING	Control	Tumor	0.037	*

Furthermore, correlations between several genes and pathways were discovered by comparing the intersection of gene expression levels and pathway scores (Fig. 2C). The gene PVT1 suggested a positive correlation with the G2/M checkpoint (*R* = 0.26, *P* = 0.036) and DNA repair (*R* = 0.26, *P* = 0.042) pathways. GOT1 showed a significant positive correlation with bile acid metabolism (*R* = 0.26, *P* = 0.039). Meanwhile, CDO1 exhibited negative correlations with G2/M checkpoint (*R* = −0.26, *P* = 0.035), PI3K/AKT/mTOR signaling (*R* = −0.26, *P* = 0.032), and interferon α response (*R* = −0.27, *P* = 0.041) pathways.

### Immune Cell Infiltration Assessment

**Fig. 3:**
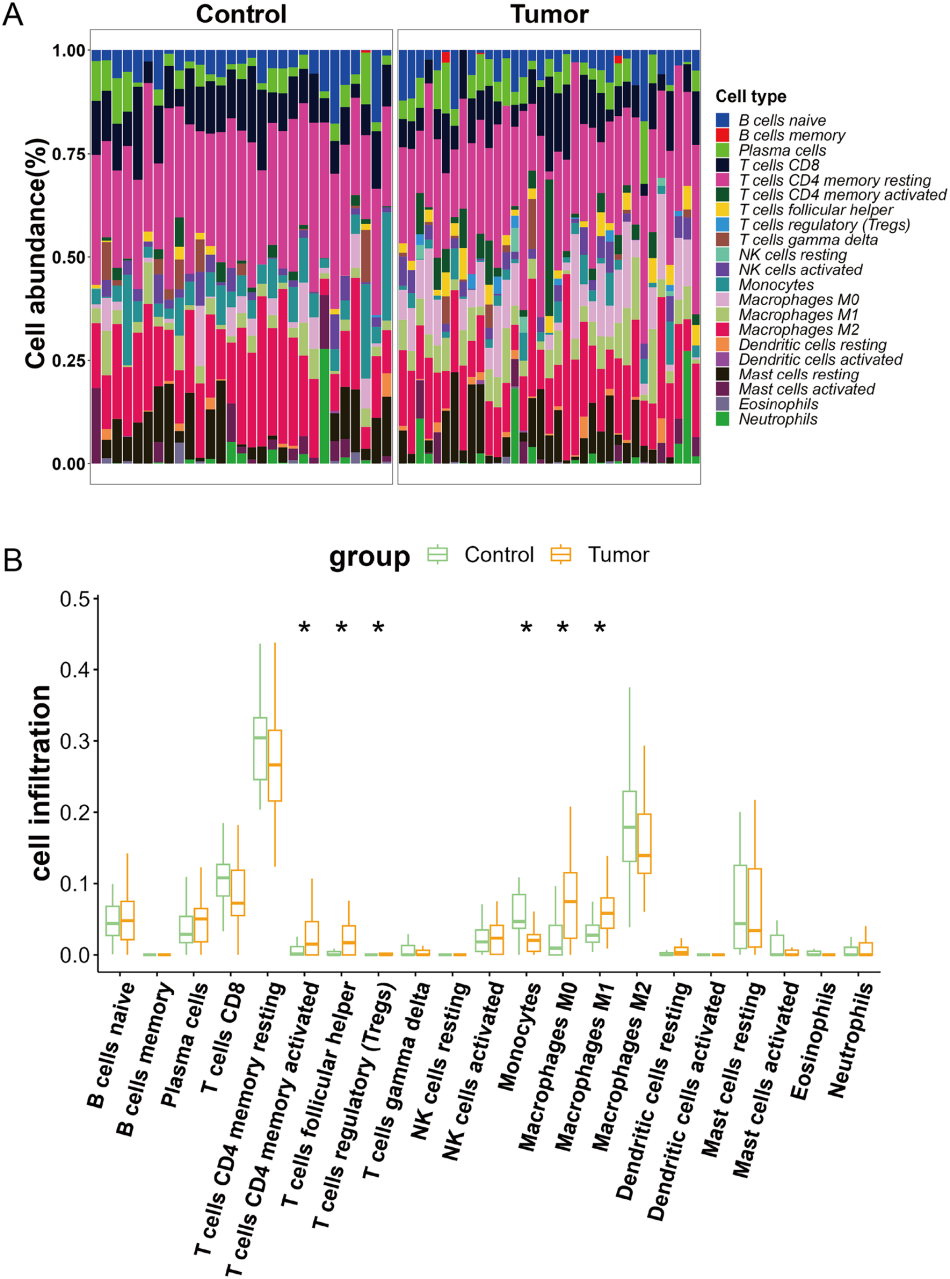
Assessment of immune cell infiltration in GBC. (**A**) Heatmap of the infiltration data for 21 types of immune cells in control and tumor groups. (**B**) Box plot shows the infiltration levels of various immune cells in the tumor group as compared to the control group; *P < 0.05

Cell types with zero values across all samples in the predicted immune cell infiltration matrix were excluded, leading to the final infiltration data for 21 types of immune cells (Fig. 3A). Differential analysis revealed that, compared to the control group, higher levels of infiltration levels of macrophages M0, macrophages M1, T cells CD4 memory activated, T cells follicular helper, and T cells regulatory (Tregs) were observed in the tumor group (*P* < 0.05, Fig. 3B). In contrast, the tumor group showed lower levels of monocyte infiltration (*P* < 0.05, Fig. 3B).

**Fig. 4:**
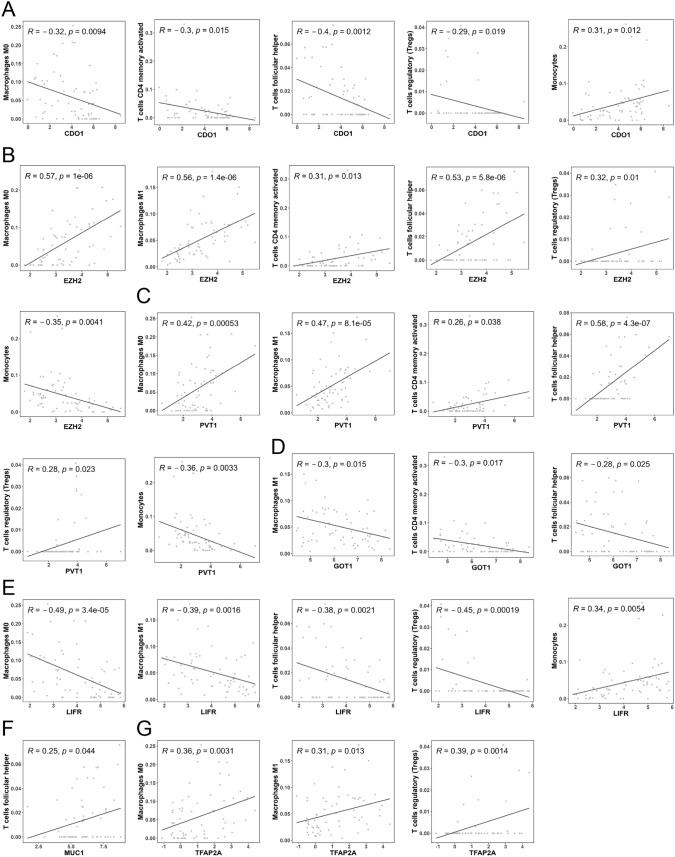
Correlation of intersecting genes and immune cell infiltration levels. (**A**) CDO1 is negative correlated with Macrophages M0, T cells memory activated, T cells follicular helper, and T cells regulatory (Tregs); and is positive correlated with Monocytes. (**B**) EZH2 was positively correlated with Macrophages M0, Macrophages M1, T cells memory activated, T cells follicular helper, and T cells regulatory (Tregs) while negatively correlated with Monocytes. (**C**) PVT1 is positively correlated with Macrophages M0, Macrophages M1, T cells memory activated, T cells follicular helper, and T cells regulatory (Tregs) while negatively correlated with Monocytes. (**D**) GOT1 is negatively correlated with Macrophages M1, T cells memory activated, and T cells follicular helper. (**E**) LIFR is negatively correlated with Macrophages M0, Macrophages M1, T cells follicular helper, and T cells regulatory (Tregs), and positively correlated with Monocytes. (**F**) MUC1 is positively correlated with T cells follicular helper. (**G**) TFAP2A is positively correlated with Macrophages M0, Macrophages M1, and T cells regulatory (Tregs)

The correlation between the expression levels of intersecting genes and the infiltration levels of immune cells were compared. We found that CDO1 showed a negative correlation with the infiltration of Macrophages M0, T cells memory activated, T cells follicular helper, and T cells regulatory (Tregs), yet a positive correlation with Monocytes (Fig. 4A); both EZH2 and PVT1 were positively related to the infiltration of Macrophages M0, Macrophages M1, T cells memory activated, T cells follicular helper, and T cells regulatory (Tregs) while showing negative relationships with Monocytes (Fig. 4B, C); GOT1 exhibited a negative correlation with the infiltration of Macrophages M1, T cells memory activated, and T cells follicular helper (Fig. 4D); LIFR displayed a negative correlation with Macrophages M0, Macrophages M1, T cells follicular helper, and T cells regulatory (Tregs), but was positively associated with Monocytes (Fig. 4E); MUC1 was positively correlated with T cells follicular helper (Fig. 4F); TFAP2A showed a positive correlation with Macrophages M0, Macrophages M1, and T cells regulatory (Tregs) (Fig. 4G).

###  Diagnostic Predictive Assessment of Ferroptosis-Related Genes

**Fig. 5:**
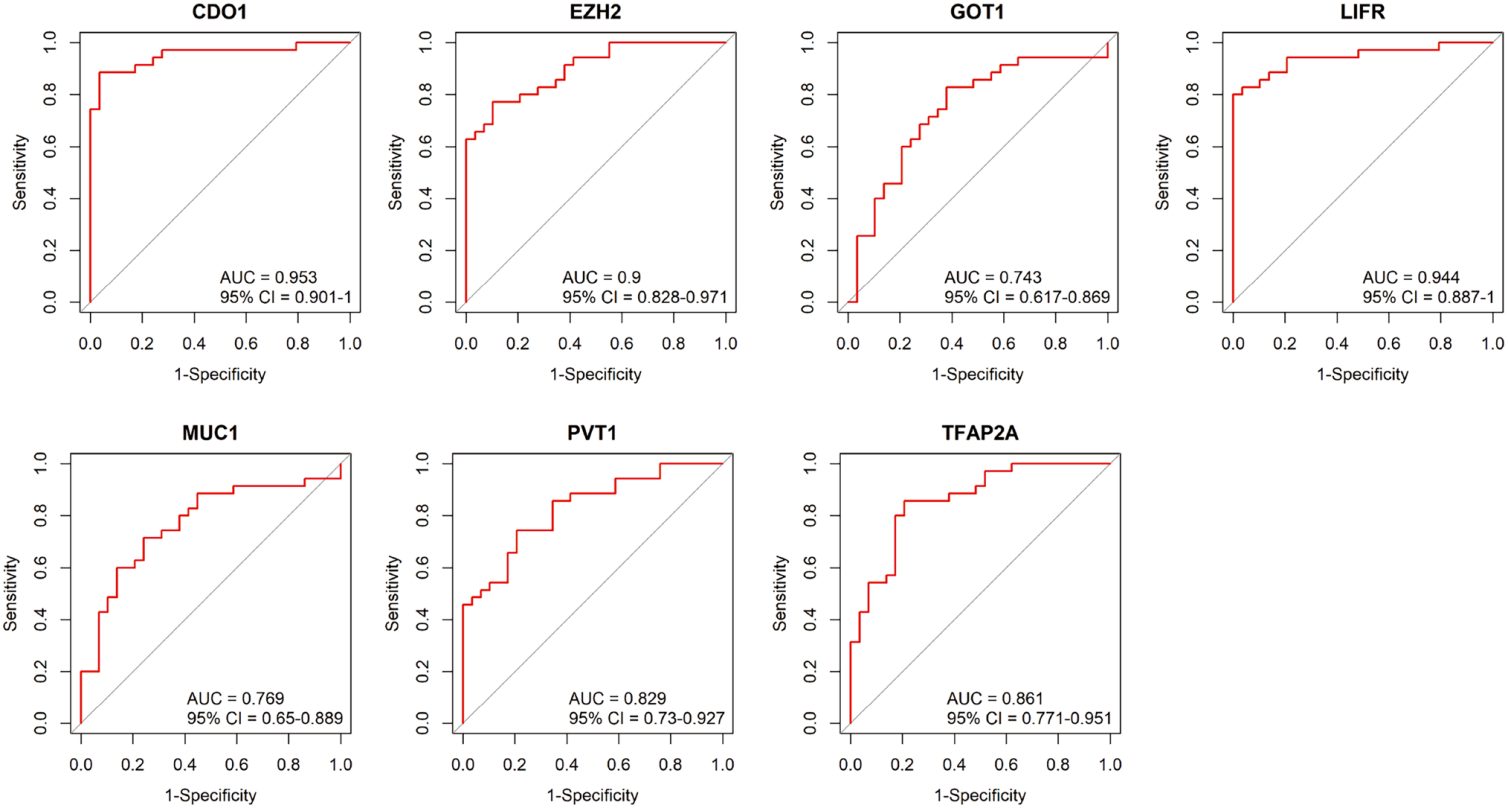
Diagnostic predictive assessment of ferroptosis-related genes. Receiver operating characteristics (ROC) demonstrate the predictive power of the seven ferroptosis-related genes (CDO1, EZH2, GOT1, LIFR, MUC1, PVT1, and TFAP2A), with the area under the curve (AUC) values displayed for each gene

We performed a univariate logistic regression analysis and assessed the predictive power of the seven ferroptosis-related genes using ROC curves. The AUC values of these genes ranged from 0.796 to 0.953 (Fig. 5), with CDO1 exhibiting the highest AUC of 0.935 (95% confidence interval: 0.901–1), EZH2 exhibiting AUC of 0.900 (95% confidence interval: 0.828–0.971), GOT1 exhibiting AUC of 0.743 (95% confidence interval: 0.617–0.869), LIFR exhibiting AUC of 0.944 (95% confidence interval: 0.887–1), MUC1 exhibiting AUC of 0.769 (95% confidence interval: 0.650–0.889), PVT1 exhibiting AUC of 0.829 (95% confidence interval: 0.730–0.927), and TFAP2A exhibiting AUC of 0.861 (95% confidence interval: 0.771–0.9510). These findings suggested that these ferroptosis-related genes hold potential value for application as diagnostic biomarkers in GBC.

## Discussion

GBC is a highly lethal malignancy with limited treatment options and a dismal prognosis, mostly attributed to its late diagnosis and rapid progression [13]. Emerging evidence has highlighted the role of ferroptosis, a novel form of regulated cell death, in various diseases, especially cancers [14]. Specifically, the dysregulation of ferroptosis is implicated in the initiation, progression, and therapy resistance of several cancers, including GBC [15]. This study seeks to identify and uncover the potential roles of ferroptosis-related genes in GBC. Furthermore, the relationships of these ferroptosis-related genes with key signaling pathways and immune cell infiltration were investigated. Finally, the diagnostic potentials of these genes in predicting disease status of patients with GBC were also emphasized. In summary, our findings illuminate the intricate connections between ferroptosis-related genes (EZH2, MUC1, PVT1, GOT1, CDO1, LIFR, and TFAP2A) and GBC, highlighting potential diagnostic markers and suggesting new avenues for treatment strategies.

Ferroptosis, an essential mechanism in the realm of cell death, plays a dual role in cancer biology [7]. When involved in the functions of several tumor suppressors, like p53 and BRCA1-associated protein 1 (BAP1), ferroptosis acts as an inherent defense against the development of cancer [8, 16]. On the contrary, the evasion of ferroptosis, promoted by oncogenic activity or oncogenic signaling, becomes a catalyst for tumor initiation, progression, metastasis, and resistance to treatment [14, 17, 18]. Thus, the roles of ferroptosis are crucial and varied in cancer dynamics. Our research pointed a significant correlation between GBC and seven ferroptosis-related genes: EZH2, MUC1, PVT1, GOT1, CDO1, LIFR, and TFAP2A. Among which, PVT1, CDO1, and LIFR were found to be ferroptosis-driving regulators, but EZH2, MUC1, GOT1, and TFAP2A were identified as ferroptosis suppressor regulators.

These genes, previously identified as crucial actors in various cancers including GBC via ferroptosis, have unique functions. In GBC, overexpression of EZH2 is reported to involve in the cancer invasion, migration, and poor progression [19]. EZH2 is a part of the Polycomb repressive complex 2 (PRC2) that controls gene expression through chromatin modification. It is implicated in tumorigenesis, and specifically, ferroptosis in hepatocellular carcinoma [20]. Moreover, EZH2 represses ferroptosis by upregulating SLC7A11 in tongue squamous cell carcinoma [21], which is coincidence with our predicting. However, the regulation role of EZH2 on ferroptosis in GBC have not been clarified. The transmembrane protein, MUC1, often found overexpressed in several cancers, and boosts cancer cell growth, survival, and invasion. In GBC, MUC1, acting as an anti-adhesion molecule, suppresses cell adhesion and promote tumor metastasis [22]. MUC1 has been shown to impede ferroptosis in esophageal squamous cell carcinoma cells [23], which also demonstrating the suppression role of MUC1 in ferroptosis. Long non-coding PVT1 is also reported to involve in the progression of GBC through miR-143/HK2 axis [24]. The depletion of RNA PVT1 quickens the ferroptosis process in liver cancer cells [25]. In the case of pancreatic cancer, GOT1 functions oppositely, inhibiting cell ferroptosis and thus promoting tumor progression [26]. Overexpression of CDO1 stimulates ferroptosis in cancer cells, acting as a potential restraint on tumor development [27]. According to Yao et al., the loss of LIFR promotes liver tumorigenesis and leads to resistance against drug-induced ferroptosis [28]. Lastly, TFAP2A reportedly plays an important role in propelling ferroptosis in GBC [15]. TFAP2A can also regulate the ferroptosis in GCB through Nrf2 pathway [29]. Although the regulation role of these genes with ferroptosis have been fully demonstrated in various cancers, while their roles in GBC is still absent except for TFAP2A.

Moreover, our study revealed that these genes are implicated in key signaling pathways, including the G2/M checkpoint, DNA repair, and PI3K/AKT/mTOR signaling. Previous studies have reported that dysregulation of these pathways could lead to tumorigenesis and cancer progression. Tumor cells frequently utilize the G2M checkpoint as a stop point in the cell cycle, permitting time for DNA damage repair [30]. The PI3K/AKT/mTOR signaling pathway, a central cellular communication route, governs basic intracellular functions [31]. It orchestrates cell proliferation, growth, metabolism, and movement [32]. Interference with this pathway has been linked to the regression of various human tumors, including ovarian [33] and gastric cancers [34], as well as GBC [35]. Our research unveiled a notable correlation between these pathways and the genes GOT1 and PVT1, suggesting they may wield a regulatory influence over GBC pathogenesis.

The roles of ferroptosis might be related to immune cell and immune response in tumor microenvironment (TME) [18]. In this study, a significant correlation between the seven key genes and immune cell infiltration in GBC were demonstrated. Recent studies have emphasized the role of tumor immune interactions in shaping the TME and influencing the clinical outcome in cancer patients [36–38]. Nakakubo et al. proved that CD4 and CD8 T cells tumor-infiltrating lymphocyte are vital factors in the prognosis of survival after surgical removal of GBC [39]. We found that EZH2, MUC1, PVT1, and TFAP2A are positively associated with macrophages and Tregs, both of which are known to play suppressive roles in the anti-tumor immune response [40]. In GBC, Tregs exhibited immunosuppressive characteristics, and macrophages played an important role in the TME [41].

Despite these significant findings, our study has some limitations. First, the analysis was performed on datasets from public databases, and our findings need to be validated in future experimental and clinical studies. Second, due to absent of roles of EZH2, MUC1, PVT1, GOT1, CDO1, and LIFR in GBC ferroptosis, the mechanisms through which the identified genes regulate ferroptosis in GBC remain to be explored. Finally, our study was unable to explore the potential therapeutic implications of modulating ferroptosis in GBC, which warrants further investigation.

## Conclusion

In conclusion, our study unravels the potential role of seven key ferroptosis-related genes: EZH2, MUC1, PVT1, GOT1, CDO1, LIFR, TFAP2A—in GBC. We demonstrated that these genes display a significant correlation with GBC, with distinct roles in the regulation of key signaling pathways, such as the G2M checkpoint, DNA repair, and PI3K/AKT/mTOR signaling. Intriguingly, these genes were also associated with varying levels of immune cell infiltration within GBC, suggesting their influence on the TME and, possibly, disease progression. This study offers the prospect of novel diagnostic and therapeutic strategies that leverage the intricate relationship between ferroptosis and GBC, with potential implications for improving patient outcomes. Also, how to develop effective therapeutic drugs related to ferroptosis-related gene in GBC is an important aspect in the further investigations.

## Data Availability

The datasets generated during and/or analysed during the current study are available from the corresponding author on reasonable request.
